# Soil microbial communities and enzyme activities in sea-buckthorn *(Hippophae rhamnoides)* plantation at different ages

**DOI:** 10.1371/journal.pone.0190959

**Published:** 2018-01-11

**Authors:** Miao Yang, Dan Yang, Xuan Yu

**Affiliations:** 1 Department of Forestry, College of Forestry, Northwest A&F University, Yangling, China; 2 Key Comprehensive Laboratory of Forestry, Shaanxi Province, Yangling, China; RMIT University, AUSTRALIA

## Abstract

The aim of this study was to assess the impact of forest age and season on the soil microbial community and enzyme activities in sea-buckthorn plantation system and to determine the relative contributions to soil microbial properties. Soil sampling was carried out in the dry season (April) and wet season (September) in four areas, including: abandoned farmland (NH), an 8-year- old plantation (young plantation, 8Y), a 13-year-old plantation (middle-aged plantation, 13Y), and an 18-year-old plantation (mature plantation, 18Y). The results showed that forest age and season have a significant effect on soil microbial community structure and enzyme activities. The total, bacterial, fungal, Gram-negative (G^+^), and Gram-positive (G^-^) PLFAs increased gradually with forest age, with the highest values detected in 18Y. All the detected enzyme activities showed the trend as a consequence of forest age. The microbial PLFAs and soil enzyme activities were higher in the wet season than the dry season. However, there were no significant interactions between forest age and season. A Correlation analysis suggested that soil microbial communities and enzyme activities were significantly and positively correlated with pH, total nitrogen (TN) and available phosphorus (AP). Season had a stronger influence on soil microbial communities than forest age. In general, sea-buckthorn plantations establishment might be a potential tool for maintaining and increasing soil fertility in arid and semi-arid regions.

## Introduction

Soil microbial communities are important components of terrestrial ecosystems. They play a critical role in organic matter decomposition and nutrient cycling of carbon, nitrogen, sulfur and phosphorus [1–3]. Therefore, any changes in the microbial communities or activities can affect soil biochemical processes and, consequently, influence soil fertility and plant growth. Generally, soil enzyme activities directly reflect metabolic requirements and available nutrients of soil microorganisms, which are important in the processing and recovery of key nutrients from detrital inputs and accumulated soil organic matter [4]. Extracellular enzymes, such as β-glucosidase, cellulose, protease, urease, and phosphatase are involved in the cycling of the key elements such as carbon, nitrogen and phosphorus [5–7]. Based on these findings, soil enzymes integrate information from the microbial status and soil physicochemical conditions. In general, more abundant PLFAs biomarkers and higher soil enzyme activities are often connected with higher levels of soil organic matter and fertility. The amounts of PLFAs were found to be positively correlated with the contents of soil organic matter, nitrogen and organic carbon [8]. Protease, urease and phosphatase were reported to have important impacts on increasing the contents of soil nitrogen and phosphorus [9]. Then again, soil enzymatic activities declined in degraded habitats and the degree of reduction was associated with the extent of habitat degradation [10]. These studies suggested that forest or plant competition is closely related to the soil microbial community structure and enzymatic activities, which are closely connected to soil organic matter and other nutrients. Thus, the studies of soil microbial communities and enzyme activities are important to understand soil recovery processes, sustainability, and quality.

Knowledge of the factors influencing soil microorganisms is fundamental for sustainable environmental management. Previous studies have demonstrated that forest age is an important factor affecting soil microbial community structure [11, 12] and enzyme activities [13, 14]. The quantity and quality of plant litter and root exudates determine the nutrient resources for soil microbial community [15]. Litter and root exudates were microbial growth-promoting substrates [16]. As plants develop, there are differences in substrate quantity and quality resulting in variations of soil microorganism. Their activity is dependent on substrate quantity and quality as well as on soil conditions, such as soil temperature, moisture, organic carbon, nitrogen, C/N ratio or soil pH. Seasonal variations will alter the soil environment especially soil moisture and temperature, which impact plant growth and soil organic matter inputs, thus affecting soil microbial communities and activities. These alterations will have consequences on litter and the soil organic matter decomposition process and turnover, which may in turn affect the stability and functioning of the ecosystem. Therefore, for studies on shifts in soil microbial communities or on mechanisms that determine microbial life in a specific forest ecosystem, it is important to account for seasonal changes. Moreover, these forest sites are in arid and semi-arid regions, which are sensitive to seasonal change. In recent years, research about the seasonal impact on soil microbial communities of different aged plantations ages has been increasing [12, 17, 18]. However, no previous integrative research has studied the interactive effect of forest age and season on soil chemical properties, microbial community and enzyme activities.

The Loess Plateau in China has arid and semi-arid regions, which are known for severe soil erosion and desertification. To mitigate or even eliminate this predicament, the Chinese government has implemented a long-term project named “Grain for Green” to convert large areas of farmland into forestland in the Loess hilly regions [19]. Studies showed that plantation forests and natural regeneration through secondary succession can restore degraded soil properties and maintain soil fertility [6, 20]. Sea-buckthorn (*Hippophae rhamnoides*) is widely planted for pure shrubwoods in the Loess Plateau as a pioneer tree species in restoring vegetation because of its ecological and economic values. On one hand, its berries, leaves and bark extract can be processed into multiple products, including oil, juice, alcoholic beverages, and jam [21]. On the other hand, it is not only fast growing, as well as frost, wind and drought resistant, but also nitrogen fixing by forming root nodule symbioses with N_2_-fixing actinomycetes in the genus *Frankia* [22]. The studies on sea-buckthorn plantations have focused largely on plant growth [23, 24], water physiology [25, 26], and soil properties [27, 28]. Little attention has been given to changes in soil microbial ecology [28]. The microbial community structure and enzyme activities in the sea-buckthorn plantation ecosystem are not well known. In addition, the interactive effects of plantation growth and season on microbial community and enzyme activities are not clear. Therefore, the objectives of this study are: (1) to investigate the effects of forest age on soil microbial community structures and enzyme activities in the dry and wet seasons, (2) to reveal the relationship among soil chemical properties, microbial community compositions, and enzyme activities, and (3) to quantify the impact of forest age and season on soil microbial communities.

## Materials and methods

### Site description

The study was carried out at the Huai Ping forest region in Shaanxi Province, Northwest China. This area is located between 34° 29′ to 34° 59′ N latitude and 107° 56′ to 108° 20′ E longitude, is part of the central–western Loess Plateau and has an altitude of approximately 1200 m above sea level. The region is characterized by a temperate continental monsoon climate. The mean annual temperature and frost-free period is 10.8°C and 210 d, respectively. The mean annual precipitation is 602 mm with dry and wet seasons. Rainy seasons are approximately from July to September, and the rainfall is approximately 313 to 367 mm, which accounts for over 50% of the mean annual precipitation. The soil is classified as cinnamon soil according to the China soil classification and code (GB/T 17296–2009).

Sea-buckthorn (*Hippophae rhamnoides*) plantations are widespread in this area. To study the dynamics of soil microbial community structures and enzyme activities after conversion of abandoned farmland to sea-buckthorn plantations, we selected three sea-buckthorn plantations at different stages of forest growth as follows: 8 years old (8Y), 13 years old (13Y), and 18 years old (18Y). Abandoned farmland was used as a control. The area of each site was approximately 10 ha. These sites were 150 m apart from one another and exhibited similar climatic characteristics, soil types, textures, and mineral compositions [29]. Prior to reforestation, they had similar management and planting history.

### Soil sampling

All soil samples were collected in the dry season (April) and wet season (September) of 2014. From each site, we randomly selected three plots (20 m × 20 m) as triplicates. The soil samples were obtained from the topsoil (0 cm to 10 cm) by using a soil corer (5 cm in diameter) after litter was excluded. Ten soil cores were randomly collected from each plot and combined to form a single sample. A total of 12 composite soil samples were obtained in this experiment. All soil samples were immediately passed through a 2 mm sieve and divided into three portions. One portion was immediately frozen at −20°C after sieving for phospholipid fatty acid (PLFA) analysis. The second portion was stored at 4°C for soil enzyme analysis. The last portion was air-dried in a cool, dry place for analyses of soil chemical properties.

### Analyses of soil chemical properties

Soil chemical characteristics were analyzed based on the method of Liu [30]. Soil pH was measured in distilled water using a 1:2.5 soil/solution ratio. Total organic carbon (TOC) was determined with a Shimadzu TOC-TN analyzer (Shimadzu Corp., Kyoto, Japan). Total nitrogen (TN) was measured using the Kjeldahl method. Total phosphorus (TP) and total potassium (TK) were digested by HNO_3_ + HClO_4_ and were measured by inductively coupled plasma-atomic emission spectrometry (ICP-AES). Available phosphorus (AP) was quantified using the molybdenum antimony colorimetric method. Available potassium (AK) was analyzed on a Perkin-Elmer flame photometer. Available nitrogen (AN) was measured with the alkaline hydrolysis diffusion method.

### PLFA analysis

PLFA analysis was performed using the methods of White et al. [31]. Lipids were extracted from 8 g dry-weight-equivalent of fresh soil from each sample in a monophasic solution of chloroform, methanol, and citrate buffer (1.0:2.0:0.8 v/v/v) [32]. Phospholipids were separated on solid-phase columns. Subsequently, the phospholipids were collected and methylated, and the resulting fatty acid methyl esters were analyzed using an Agilent 6850N gas chromatographer (Agilent Technologies, Palo Alto, USA) equipped with an ULTRA-2 column (25 m length × 0.20 mm internal diameter, 0.33 μm film thickness). Peaks were identified based on retention time and mass spectral information. Quantification of each PLFAs was achieved by comparing the peak areas with the internal standard methyl nonadecanoate (19:0) [33]. PLFA-values were reported as nmol g^−1^ soil. Biomarkers for the soil microbial community [34–37] are shown in Table 1. Other PLFAs detected in the current experiment, such as 10 me17:0, 18:1w7c, and 20:1w9c, were used to assess microbial compositions. The ratios of the sum of saturated (SAT) and mono-unsaturated (MONO) PLFAs were used as physiological or nutritional stress indicators [17]. For differences in PLFA composition and individual PLFAs between forest age and season, we used log-transformed mole percentages of total PLFAs within a sample.

**Table 1:** Biomarkers for soil microbial community.

	Diagnostic fatty acids
Bacteria	14:0, 15:0, 16:0, 18:0, 14:0i, 15:0i, 15:0a, 16:0i, 17:0i, 17:0a, 14:1w5c, 15:1w6c, 16:1w7c, cy17:0, cy19:0, 15:03OH, 16:12OH, 16:1w9c, 18:1w5c, cy18:0 [34,35]
Gram-positive bacteria	14:0i, 15:0i, 15:0a, 16:0i, 17:0i, and 17:0a [34]
Gram-negative bacteria	14:1w5c, 15:1w6c, 16:1w7c, cy17:0, cy19:0, 15:03OH, 16:12OH, 16:1w9c, 18:1w5c, cy18:0[34]
Fungi	18:1w9c and 18:3w6c [36,37]

### Soil enzyme activities

The activities of five extracellular enzymes were determined based on the methods of Guan [38]. Five gram soil samples were used for alkaline phosphatase and urease analysis. Another three samples of one gram of soil were used to determine the activities of β-glucosidase, cellulose, and protease. The substrates were disodium phenylphosphate, urea, p-nitrophenyl-β-D-glucoside, carboxymethyl cellulase and casein. Distilled water was used as the substrates for controls. All five enzyme activities were assayed via the colorimetric method. The results were expressed based on soil dry matter. Three replicates for each enzyme were analyzed.

### Data analysis

The effects of forest age, season and interactions on soil chemical properties, microbial community structures and enzyme activities were determined among the tested soil samples from the four sites by using two-way ANOVA. If the differences were significant, a pair-wise Fisher’s least significant difference (LSD) test was performed to determine where the difference is. Pearson correlation and redundancy analysis (the vegan package of R-language) were performed to examine the relationships among soil chemical properties, microbial community compositions, and enzyme activities. Principal component analysis (PCA) was used to analyze the changes of PLFA profiles and enzyme activities related to forest age. Discriminant analysis was also applied to the PLFA profiles to detect the patterns at different forest ages and seasons. Discriminant function (DF) scores were plotted to show how forest ages and seasons were clustered. The above analyses were performed with SPSS (version 19.0). In addition, partial redundancy analyses were used to quantify the contribution of forest age and season on soil microbial communities under the vegan package of the R-language environment (version 2.9, R Foundation for Statistical Computing, Vienna, Austria). All of the data were tested for normal distribution and homogeneity of variance, respectively. The mean of each PLFA was log transformed to create a more normally distributed data set and to reduce the coefficient of variation among PLFAs.

## Results

### Soil chemical properties

As shown in Table 2, forest age had a significant effect on soil chemical properties. The contents of TOC, TN, AP, and AN increased gradually with forest age. The maximum contents of these parameters, based on the average values of two seasons, were observed in 18Y and increased significantly by 146.3%, 52.2%, 24.9%, and 3.5% compared with those in 8Y, respectively. However, the highest contents of soil TK and AK were measured in 8Y. In contrast, soil TK and AK decreased significantly in 13Y and increased slightly in 18Y. There are no significant differences for soil TK and AK between 13Y and 18Y. Soil pH increased significantly after conversion from abandoned farmland to sea-buckthorn plantation. As forest age increased, soil pH increased gradually, and the highest values were found in 18Y. In addition, significant difference among seasons was observed in all plantation stands when examined individually. All of the soil properties were higher in the wet season compared to dry season. There were no significant interactions between forest age and season.

**Table 2:** Effects of forest age, season and their interactions on soil chemical properties (means±s.d., n = 3).

Factors	pH	TOC^d^ (g kg^-1^)	TN (g kg^-1^)	TP (g kg^-1^)	TK (g kg^-1^)	AP (mg kg^-1^)	AK (mg kg^-1^)	AN (mg kg^-1^)
FA^a^								
*P*-value	0.002	<0.001	<0.001	<0.001	0.005	<0.001	<0.001	<0.001
NH^b^	8.51±0.12a^c^	6.37±0.79a	1.12±0.43a	0.21±0.07a	11.75±0.98a	5.76±0.74a	101.08±2.73a	85.90±3.64a
8Y	8.56±0.14b	6.53±0.84a	1.15±0.31a	0.24±0.10a	14.50±1.45c	6.06±0.65a	106.69±1.87b	86.26±3.70a
13Y	8.61±0.12b	14.86±1.56b	1.44±0.34b	0.32±0.14a	12.62±1.38ab	7.13±0.98b	102.58±2.57a	87.90±3.89b
18Y	8.66±0.18c	16.08±1.04c	1.75±0.53c	0.63±0.19b	14.09±1.92bc	7.57±0.92c	103.41±2.16a	89.31±4.43c
S								
*P*-value	<0.001	<0.001	0.001	0.028	0.005	<0.001	<0.001	<0.001
DS	8.52±0.13a	10.60±4.76a	1.26±0.43a	0.29±0.18a	12.41±1.32a	5.97±0.79a	101.80a	83.88±1.50a
WS	8.64±0.14b	11.32±4.92b	1.49±0.48b	0.41±0.23b	14.07±1.83b	7.29±0.94b	105.32b	90.81±1.91b
FA×S								
*P*-value	0.684	0.740	0.120	0.457	0.785	0.118	0.960	0.638

^a^ FA: forest age; S: season; DS: dry season; WS: wet season; FA×S: interactions of forest age and season.

^b^ NH: abandoned farmland; 8Y: 8 years old; 13Y: 13 years old; 18Y: 18 years old.

^c^ Different letters in the same columns indicate significant differences at *P* < 0.05 levels via LSD test.

^d^ TOC: total organic carbon; TN: total nitrogen; TP: total phosphorus; TK: total potassium; AP: available phosphorus; AK: available potassium; AN: available nitrogen.

### Microbial community structure

Biomass of Gram-positive (G^+^) bacteria, Gram-negative (G^−^) bacteria, and bacteria in general increased significantly after conversion from abandoned farmland to sea-buckthorn plantation (Table 3). Nevertheless, there were no significant differences in total PLFAs and fungal PLFAs between NH and 8Y. As forest age increased, all of the above parameters also increased gradually. The amount of total PLFAs, G^-^PLFAs, and bacterial PLFAs were significantly higher in 13Y and 18Y than in 8Y. Seasons also had significant effects on the soil microbial community. However, the interaction between forest age and season was not significant for the soil microbial community structure.

**Table 3:** Effects of forest age, season and their interactions on soil microbial communities (means±s.d., n = 3).

Factors	Total lipid	G^+^^d^	G^-^	Bacteria	Fungi	AMF	S/M	G^+^/G^-^	F/B
FA^a^									
P-value	<0.001	<0.001	<0.001	<0.001	0.05	<0.001	0.05	0.028	<0.001
NH^b^	13.91±4.68a^c^	3.17±1.54a	1.43±0.60a	7.65±3.30a	1.40±0.44a	0.71±0.40a	1.30±0.23a	2.19±0.46a	0.19±0.04a
8Y	16.40±3.20a	4.27±1.59bc	2.19±0.60b	10.01±2.87b	1.61±0.39ab	0.84±0.09ab	1.21±0.07ab	1.90±0.25ab	0.16±0.02b
13Y	20.43±3.68b	5.09±1.85cd	2.97±0.90c	12.41±3.80c	1.73±0.43b	1.04±0.08ab	1.21±0.06ab	1.71±0.46ab	0.14±0.02c
18Y	21.32±4.27b	5.48±2.05d	3.37±0.82c	12.94±3.84c	1.84±0.58b	1.16±0.05b	1.07±0.04b	1.58±0.30b	0.14±0.01c
S									
*P*-value	<0.001	<0.001	<0.001	<0.001	<0.001	<0.001	0.004	0.006	0.003
DS	14.74±3.70a	2.98±1.88a	1.94±0.80a	7.82±2.32a	1.28±0.25a	0.73±0.18a	1.09±0.14a	1.63±0.42a	0.17±0.04b
RS	21.29±3.41b	6.02±1.21b	3.04±0.96b	13.68±2.74b	2.01±0.31b	1.15±0.41b	1.31±0.20b	2.06±0.31b	0.15±0.01a
FA×S									
*P*-value	0.742	0.691	0.984	0.837	0.754	0.314	0.480	0.874	0.114

^a^ FA: forest age; S: season; DS: dry season; WS: wet season; FA×S: interactions of forest age and season.

^b^ NH: abandoned farmland; 8Y: 8 years old; 13Y: 13 years old; 18Y: 18 years old.

^c^ Different letters in the same columns indicate significant differences at *P* < 0.05 levels via LSD test.

^d^ G^+^: gram-positive bacteria; G^-^: gram-negative bacteria; S/M: the ratio of saturated to monounsaturated fatty acids; G^+^/ G^-^: the ratio of the sum of gram-positive bacteria to the sum of gram-negative bacteria; F/B: the ratio of fungi to bacteria.

**Fig 1:**
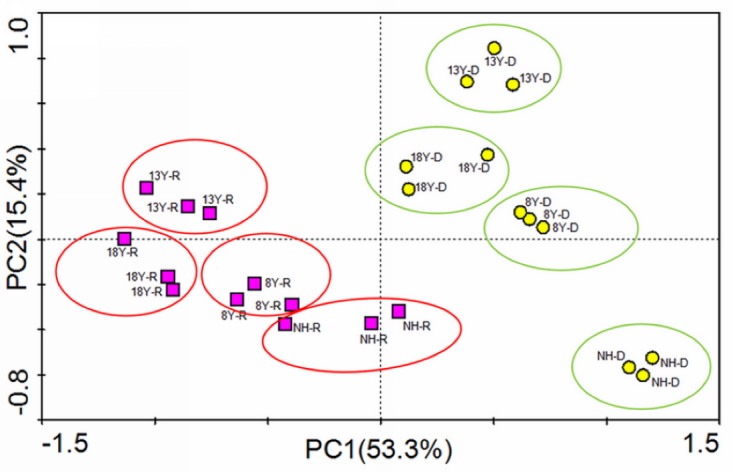
PCA of fatty acids from the sites of different ages in dry and wet season. NH-R: abandoned farmland in wet season; 8Y-R: 8 years old plantation in wet season; 13Y-R: 13 years old plantation in wet season; 18Y-R: 18 years old plantation in wet season; NH-D: abandoned farmland in dry season 8Y-D: 8 years old plantation in dry season; 13Y-D: 13 years old plantation in dry season; 18Y-D: 18 years old plantation in dry season.

Based on the relative abundance of total PLFAs, PCA showed a clear discrimination among the structures of soil microbial communities associated with different forest ages and seasons (Fig 1). Components 1 and 2 explained 53.3% and 15.4% of the variation, respectively. In component 1, fatty acids 15:0i, 15:0a, 16:0i, 17:0i, 17:0a, 17:0cyclo, 16:12OH, 18:1w5c, 19:0cyclow8c, 16:0, 18:0, 16:0a, 16:1w5c, 18:1w9c, and 10me18:0 exhibited high factor weights. In component 2, 14:0i, 16:1w7c, 17:1w8c, 18:03OH, 20:1w9c, and 20:0 also showed high factor weights.

**Fig 2:**
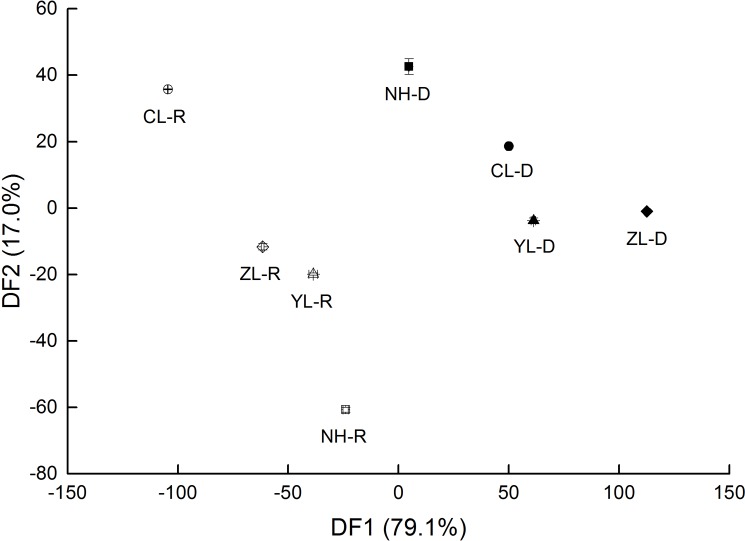
Ordination plot of discriminant functions based on different forest ages and seasons. NH-R: abandoned farmland in wet season; 8Y-R: 8 years old plantation in wet season; 13Y-R: 13 years old plantation in wet season; 18Y-R: 18 years old plantation in wet season; NH-D: abandoned farmland in dry season 8Y-D: 8 years old plantation in dry season; 13Y-D: 13 years old plantation in dry season; 18Y-D: 18 years old plantation in dry season.

Discriminant analysis showed that the first two axes could explain 79.1%and 17.0%of the total variation in microbial communities, respectively (Fig 2). Seasons were separated from each other on the first discriminant function (DF) axis. On the second axis, the microbial communities at different forest ages were distinctly separated from each other.

### Enzyme activities

Forest age had a significant effect on all the soil enzyme activities determined in this study (Table 4). There were no significant differences in the activities of β-glucosidase, cellulose, urease and protease between NH and 8Y. However, the phosphatase activity was higher in 8Y than that in NH. All the five enzyme activities increased gradually with forest age and the highest values were presented in 18Y. A significant seasonal effect was detected for all enzyme activities, showing higher enzyme production in the wet season than in the dry season. However, the interaction between plantation age and season were not significant.

**Table 4:** Effect of forest age and season on soil enzyme activities (means±s.d., n = 3).

Factors	β-glucosidase (ug g^-1^dw h^-1^)	Cellulase (mg g^-1^dw h^-1^)	Urease (mg g^-1^dw d^-1^)	Protease (mg g^-1^dw h^-1^)	Phosphatase (mg g^-1^dw d^-1^)
FA^a^					
*P*-value	<0.001	<0.001	<0.001	<0.001	<0.001
NH^b^	43.86±1.15a^c^	0.30±0.19a	0.26±0.13a	0.13±0.09a	0.32±0.09a
8Y	45.00±1.33a	0.38±0.18a	0.32±0.15a	0.16±0.09a	0.53±0.14b
13Y	46.56±2.06b	0.54±0.19b	0.48±0.16b	0.42±0.16b	0.73±0.09c
18Y	47.87±2.52b	0.62±0.13b	0.66±0.14c	0.56±0.14c	0.80±0.09c
S					
*P*-value	<0.001	0.001	<0.001	<0.001	<0.001
DS	44.53±1.56a	0.41±0.21a	0.37±0.20a	0.32±0.19a	0.58±0.21a
RS	47.12±2.27b	0.50±0.21b	0.47±0.22b	0.44±0.24b	0.67±0.20b
FA×S					
*P*-value	0.085	0.258	0.766	0.414	0.972

^a^ FA: forest age; S: season; DS: dry season; WS: wet season; FA×S: the interactions of forest age and season

^b^ NH: abandoned farmland; 8Y: 8 years old; 13Y: 13 years old; 18Y: 18 years old.

^c^ Different letters in the same columns indicate significant differences at *P* < 0.05 levels via LSD test.

### Correlations among soil chemical properties, microbial community structures, and enzyme activities

**Fig 3:**
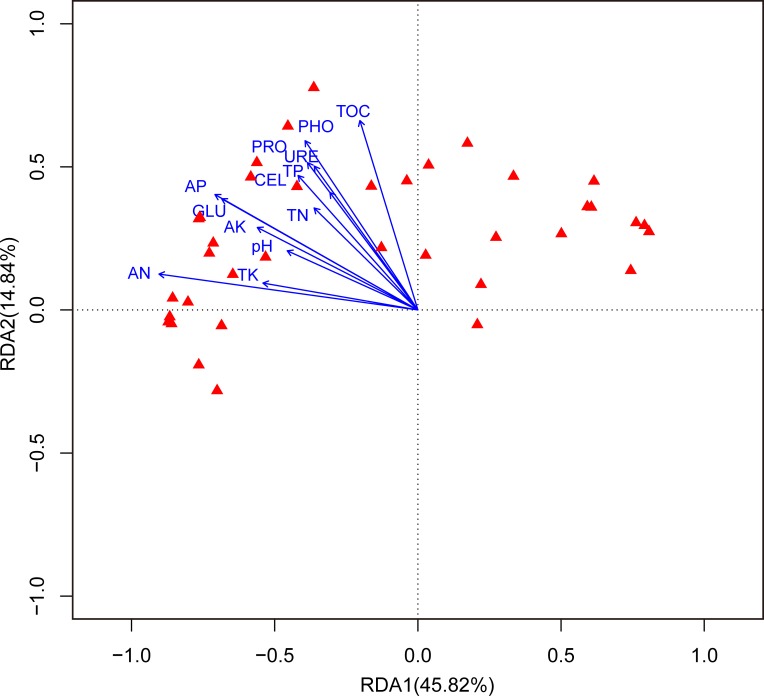
Redundancy analysis on microbial communities and soil properties. The solid red triangle presents for individual PLFA determined in sites. TOC: total organic carbon; TN: total nitrogen; TP: total phosphorus; TK: total potassium; AP: available phosphorus; AK: available potassium; AN: available nitrogen. GLU: β-glucosidase; CEL: Cellulase; URE: Urease; PRO: protease; PHO: phosphatase.

The Pearson’s correlations analysis revealed that total PLFAs, bacterial PLFAs, G^+^PLFAs, and G^-^PLFAs were significantly correlated with pH, TOC, TN, TP, AN, and AP (Table 5). However, the above microbial variables showed no significant correlations with TK and AK. In addition, the correlation coefficients between the soil properties and enzyme activities are shown in Table 5. All of the detected enzyme activities were significantly correlated with pH, TOC, TN, and AP. RDA showed that the first axis and the second axis accounted for 45.82% and 14.84% of the total variation, respectively (Fig 3). Forward selection of environmental variables showed that soil TOC, AN, urease, and β-glucosidase significantly affected the microbial community structure.

**Table 5:** Correlation coefficients between soil chemical properties and microbial community and enzyme activity.

	pH	TOC^a^	TN	TP	TK	AN	AP	AK
Total lipids	0.460*	0.661**	0.618**	0.672**	0.287	0.824**	0.893**	0.060
Bacteria	0.477*	0.567**	0.611**	0.620**	0.334	0.867**	0.894**	0.150
Fungi	0.431*	0.359	0.514*	0.499*	0.288	0.836**	0.766**	0.188
G^+^	0.515*	0.493*	0.601**	0.602*	0.370	0.900**	0.869**	0.195
G^-^	0.419*	0.733**	0.625**	0.713**	0.360	0.724**	0.892**	0.134
GLU^b^	0.427*	0.652**	0.513*	0.696**	0.480*	0.756**	0.898**	0.566**
CEL	0.447*	0.610**	0.434*	0.396	0.147	0.392	0.563**	0.421*
URE	0.461*	0.719**	0.405*	0.638**	0.342	0.476*	0.638**	0.351
PRO	0.465*	0.849**	0.696**	0.685**	0.173	0.554**	0.777**	0.500*
PHO	0.470*	0.825**	0.527**	0.617**	0.375	0.539**	0.774**	0.636**

^a^ TOC: total organic carbon; TN: total nitrogen; TP: total phosphorus; TK: total potassium; AP: available phosphorus; AK: available potassium; AN: available nitrogen.

^b^ GLU: β-glucosidase; CEL: Cellulase; URE: Urease; PRO: protease; PHO: phosphatase.

* denotes significant differences at *P* < 0.05.

** denotes significant differences at *P* < 0.01.

**Fig 4:**
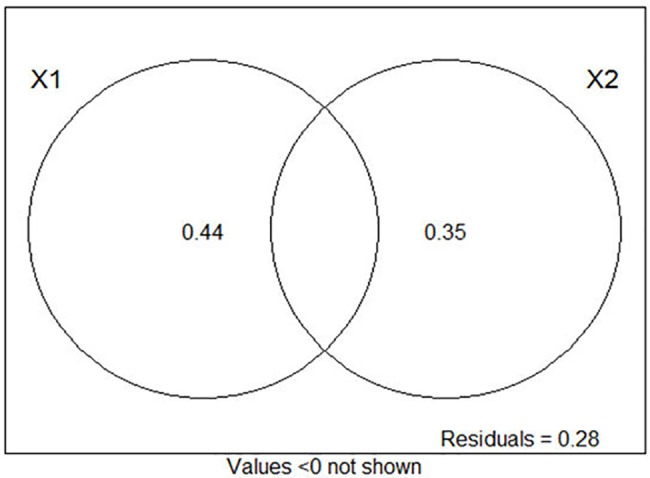
Partial RDA for forest age, season and soil micorbial communities. X1: season; X2: forest age.

In addition, the results of the partial redundancy analysis showed that season explained 44.0% of soil microbial communities. However, forest age explained 35.0% of soil microbial communities (Fig 4). Therefore, season had the greater impacts on soil microbial communities.

## Discussion

The vegetation cover has an important effect on soil properties [39] through its contribution of organic matter inputs from plant residues and root exudates to the soil. In the present work, as forest age increased, the contents of TOC, TN, and AN increased gradually and the highest values were found in the 18Y plantation. This finding suggested significant improvements in soil physicochemical properties resulted from plantation growth and the positive effects increased with plantation age. Carbon and N accumulations have been regarded as indicators of soil fertility and productivity [6]. Sea-buckthorn plantation succession is useful for improvement of soil fertility.

Generally, soil microbial biomass or microbial PLFA abundance has traditionally been used as an indicator of soil fertility, with changes of arbuscular mycorrhizal fungal (AMF) PLFA, the ratios of saturated to monounsaturated fatty acids, G^+^ to G^-^ bacteria, and fungal to overall bacterial PLFA (F/B) indicating variation in soil quality [40–42]. In our study, the PLFA abundances increased gradually with plantation age, which demonstrated that soil fertility recovered gradually after reforestation. Furthermore, individual PLFA signatures, including arbuscular mycorrhizal fungal (AMF) PLFA, might be sensitive indicators of improvement in soil abiotic conditions [17]. Our results showed that the AMF PLFA 16:1w5c increased gradually with the plantation growth and the highest value was detected in 18Y. AMF are known to play an important role in enhancing the sustainability of ecosystems by improving soil structure [43, 44]. The high values of AMF PLFA in 18Y plantations confirmed that soil quality improved after reforestation. Our results showed that the ratios of saturated to monounsaturated fatty acids and G^+^ to G^-^ bacteria, which positively correlated with soil nutritional stress or negatively with resource availability [17], decreased with the growth of the sea-buckthorn plantation. The decreases of the ratios imply increased soil resource availability or decreased soil nutrient stress. Therefore, the soil quality had been improved gradually with the increasing forest age. In addition, our results showed that the ratio of fungal to bacterial PLFA (F/B) decreased and the bacteria increased gradually as the forest age increased. The ratio was low compared to previous studies [45]. It suggested that bacteria predominated in our sampling sites. We speculate that the bacterial communities recovered faster and had higher efficient dispersal and colonizing abilities than the soil fungal communities. On the other hand, this result might be correlated with the fast decomposition of forest litter.

Soil microorganisms excrete soil enzymes to drive mineralization and decomposition. These microorganisms are directly responsible for the initial processing of nutrient cycling and the variation of vegetation communities. Therefore, soil enzyme activities may be related to the element cycling in soil. The results showed that the activities of urease, protease, and phosphatase were significantly higher at 13Y and 18Y than at 8Y and NH. This suggests that plant growth may accelerate the mineralization rates of soil organic nitrogen and phosphorus and further increase the content of soil nitrogen and phosphorus. Enzymatic activities usually have been tested as the indices of site fertility [46]. In this study, we found that all the detected enzyme activities increased gradually with forest age. We deduced that soil fertility increased in the sea-buckthorn plantations. In addition, it is evident that soil enzyme activities can potentially predict disturbance and stress that the soil microbial communities suffered [47]. Furthermore, enzymatic activities increased with increasing soil organic C along the plantation chronosequence due to the dependence of microbial activity on the supply of substrate C [6]. Our data indicates that all of the five enzyme activities increased gradually, and the highest values were presented in 18Y. This can be explained as follows: the microbial community at 18Y faces less stress than those at the other sites because of richer resources. The higher soil organic C can provide enough substrate to support higher microbial biomass, hence higher enzyme production.

In general, seasonal variations of soil microbial communities were in accordance with the changes of soil moisture and temperature [48]. Our results showed that seasonal variations have important impacts on soil microbial communities and enzyme activities. There were different microbial compositions between the dry and wet season. In the dry season, low soil moisture content was not fit for the growth of soil microorganisms. Furthermore, it limited plant growth and decreased the supply of available substrate, which explains the lower abundance of the different groups of microorganisms. Soil enzyme activities are the direct expressions of the soil microbial community to metabolic requirements and available nutrients [49]. Slow microbial growth would decrease soil enzyme activities. However, some studies have different opinions on the effect of seasonal shifts. Lipson et al. suggested that in an alpine dry meadow, soil microbial biomass was higher when soil moisture was lower [50]. Some researchers reported that seasonal shifts have no or little impact on soil microbial properties in different regions [51]. These studies suggest there is no clear seasonal pattern on soil microbial properties in forest ecosystems. The seasonal variation of soil microbial properties may be closely related to biotic and abiotic factors in specific region, such as vegetation type, growth, soil nutritional conditions, temperature, water availability, proton concentration, and oxygen supply. Therefore, specific environmental conditions, especially climate conditions and habitat should be considered in further studies.

Plant species, diversity, vegetation, and forest type altered soil microbial community structure because the differences in litter quantity and quality can affect the formation of soil organic matter, which provides nutrient substrates for microbial growth [52, 53]. The present study showed that forest age has a significant effect on soil chemical properties. Total PLFAs, G^+^ bacterial biomass, G^-^ bacterial biomass, and overall bacterial biomass exhibited significant positive correlations with soil pH, TOC, TN, TP, AN, and AP. This result suggested that soil chemical properties were closely related to soil microbial community structure. Plant growth indirectly affects the soil microbial community through soil chemical properties. This finding was consistent with those of many previous studies [54, 55]. In addition, the Loess Plateau is in an arid and semi-arid region. The previous study demonstrated that seasonal variations of soil microbial communities and activities were caused by the changes of soil moisture and substrate availability [56]. Compared with other regions, soil moisture and temperature could noticeably change in different seasons and lead to significant differences in aboveground plant growth and diversity. Therefore, it is assumed that in this region, season is a crucial factor, which might affect plant growth and substrate availability and indirectly affect soil microbial communities, as demonstrated by the partial redundancy analysis (Fig 4). Changes in soil properties and microbial activities might affect plant growth and the effect of restoration. To some extent, evaluation of seasonal shifts in soil chemical properties, microbial communities and enzyme activities were useful in understanding changes in soil environment and fertility, which may help guide selection of plant species and sowing time.

## Conclusion

Plantation growth or season has a significant effect on soil microbial communities and enzyme activities. The amounts of soil microbial PLFAs and enzyme activities increased gradually with forest age and positively correlated with soil pH, TOC, TN, TP, AN, and AP, indicating that substrate availability significantly influenced the soil microbial community and function. There was an increase in these parameters in the wet season compared to the dry season during plantation development. In the detected region, partial redundancy analysis (Fig 4) showed that season had a stronger influence on soil microbial communities and enzyme activities than plantation growth. Season might affect plant growth and substrate availability; thereby indirectly affect soil microbial properties. In addition, since sea-buckthorn is a nitrogen-fixing plant, thus having the ability to increase soil fertility, plantations might be a potential tool for restoration of ecological function in the degraded temperature ecosystem of northwestern China.

## Supporting information

S1 File The relevant data including soil chemical properties, microbial communities and enzyme activities in different seasons. (DOC)
