# Supplementary material for: Soil microbial communities and enzyme activities in sea-buckthorn (Hippophae rhamnoides) plantation at different ages
Source: PLoS One. 2018 Jan 11;13(1):e0190959. doi: 10.1371/journal.pone.0190959 (PMC5764322; doi:10.1371/journal.pone.0190959)
Supplement: S1 File — (DOC) [file pone.0190959.s001.doc]

S1 File

Supporting Information

**Table A. Soil chemical properties in dry season.**

| Sites | pH | TOC | TN | TP | TK | AP | AK | AN |
| --- | --- | --- | --- | --- | --- | --- | --- | --- |
| NH | 8.55 | 6.71 | 1.60 | 0.11 | 12.22 | 5.77 | 98.67 | 83.32 |
| NH | 8.31 | 6.09 | 0.56 | 0.27 | 10.66 | 4.63 | 97.34 | 81.91 |
| NH | 8.52 | 5.34 | 1.05 | 0.16 | 11.07 | 5.16 | 102.15 | 82.76 |
| 8Y | 8.68 | 6.03 | 0.83 | 0.23 | 13.76 | 5.03 | 98.76 | 83.62 |
| 8Y | 8.34 | 7.18 | 1.16 | 0.10 | 13.19 | 5.91 | 102.34 | 81.35 |
| 8Y | 8.52 | 5.56 | 1.44 | 0.33 | 13.87 | 5.75 | 100.46 | 84.07 |
| 13Y | 8.56 | 12.91 | 0.83 | 0.23 | 13.07 | 6.84 | 106.56 | 84.32 |
| 13Y | 8.63 | 14.38 | 1.28 | 0.11 | 10.98 | 5.79 | 104.35 | 85.67 |
| 13Y | 8.44 | 16.15 | 1.64 | 0.42 | 11.35 | 6.33 | 105.97 | 83.39 |
| 18Y | 8.58 | 15.43 | 1.11 | 0.55 | 14.76 | 6.41 | 103.35 | 85.35 |
| 18Y | 8.41 | 16.66 | 2.16 | 0.68 | 11.70 | 7.33 | 101.09 | 84.27 |
| 18Y | 8.75 | 14.74 | 1.43 | 0.31 | 12.29 | 6.71 | 100.56 | 86.48 |

**Table B. Soil chemical properties in wet season.**

| Sites | pH | TOC | TN | TP | TK | AP | AK | AN |
| --- | --- | --- | --- | --- | --- | --- | --- | --- |
| NH | 8.60 | 5.94 | 1.65 | 0.22 | 11.09 | 6.25 | 103.45 | 89.28 |
| NH | 8.42 | 7.68 | 0.97 | 0.30 | 12.21 | 6.63 | 100.56 | 90.12 |
| NH | 8.63 | 6.43 | 0.86 | 0.18 | 13.26 | 6.13 | 104.32 | 88.01 |
| 8Y | 8.56 | 6.12 | 0.79 | 0.24 | 15.83 | 6.19 | 105.25 | 88.78 |
| 8Y | 8.73 | 6.43 | 1.35 | 0.17 | 13.57 | 6.81 | 104.89 | 90.21 |
| 8Y | 8.52 | 7.85 | 1.52 | 0.38 | 16.79 | 6.68 | 103.78 | 89.53 |
| 13Y | 8.67 | 17.24 | 1.49 | 0.40 | 13.23 | 7.64 | 109.56 | 92.42 |
| 13Y | 8.53 | 14.39 | 1.78 | 0.47 | 12.35 | 8.35 | 106.45 | 90.32 |
| 13Y | 8.80 | 14.11 | 1.61 | 0.28 | 14.76 | 7.84 | 110.23 | 91.25 |
| 18Y | 8.55 | 15.88 | 1.32 | 0.66 | 15.15 | 8.24 | 105.12 | 92.31 |
| 18Y | 8.91 | 16.04 | 2.09 | 0.77 | 16.88 | 8.78 | 104.56 | 93.01 |
| 18Y | 8.76 | 17.77 | 2.41 | 0.83 | 13.77 | 7.95 | 105.78 | 94.43 |

**Table C. Soil microbial communities in dry season**.

| Sites | TPLFA | G^+^ | G^-^ | BPLFA | FPLFA | AMF |
| --- | --- | --- | --- | --- | --- | --- |
| NH | 8.82 | 1.51 | 1.07 | 4.56 | 1.03 | 0.51 |
| NH | 10.50 | 1.80 | 0.90 | 4.73 | 1.12 | 0.59 |
| NH | 10.44 | 2.30 | 0.83 | 5.36 | 1.13 | 0.62 |
| 8Y | 13.03 | 2.66 | 1.61 | 7.08 | 1.42 | 0.56 |
| 8Y | 13.74 | 2.82 | 1.80 | 7.61 | 1.29 | 0.55 |
| 8Y | 13.88 | 3.01 | 1.57 | 7.60 | 1.12 | 0.77 |
| 13Y | 16.44 | 3.20 | 2.35 | 8.67 | 1.44 | 0.78 |
| 13Y | 17.63 | 3.29 | 2.34 | 9.06 | 1.42 | 0.83 |
| 13Y | 18.33 | 3.82 | 2.53 | 9.65 | 1.22 | 0.85 |
| 18Y | 15.09 | 2.42 | 2.06 | 6.97 | 0.87 | 0.61 |
| 18Y | 21.19 | 4.80 | 3.43 | 12.29 | 1.82 | 1.04 |
| 18Y | 17.76 | 4.14 | 2.82 | 10.27 | 1.45 | 1.01 |

**Table D. Soil microbial communities** in wet season.

| Sites | TPLFA | G^+^ | G^-^ | BPLFA | FPLFA | AMF |
| --- | --- | --- | --- | --- | --- | --- |
| NH | 20.71 | 5.46 | 2.39 | 12.70 | 2.21 | 1.28 |
| NH | 16.99 | 3.99 | 1.75 | 9.60 | 1.50 | 1.40 |
| NH | 15.97 | 3.93 | 1.62 | 8.97 | 1.40 | 1.16 |
| 8Y | 19.97 | 5.66 | 2.87 | 12.66 | 2.11 | 1.13 |
| 8Y | 18.09 | 5.42 | 2.50 | 11.80 | 1.76 | 0.95 |
| 8Y | 19.72 | 6.03 | 2.81 | 13.29 | 1.96 | 1.08 |
| 13Y | 24.10 | 6.71 | 4.03 | 16.08 | 1.96 | 1.20 |
| 13Y | 25.51 | 7.31 | 4.23 | 17.36 | 2.29 | 1.43 |
| 13Y | 20.59 | 6.19 | 2.36 | 13.63 | 2.08 | 1.16 |
| 18Y | 23.25 | 6.54 | 3.93 | 15.43 | 2.34 | 1.71 |
| 18Y | 26.77 | 7.76 | 4.38 | 17.18 | 2.25 | 1.39 |
| 18Y | 23.83 | 7.21 | 3.59 | 15.48 | 2.30 | 1.21 |

**Table E**. Soil enzyme activities in dry season.

| Sites | β-glucosidase | Cellulase | Urease | Protease | Phosphatase |
| --- | --- | --- | --- | --- | --- |
| NH | 42.03 | 0.13 | 0.20 | 0.20 | 0.15 |
| NH | 44.31 | 0.22 | 0.14 | 0.04 | 0.36 |
| NH | 43.08 | 0.50 | 0.41 | 0.10 | 0.29 |
| 8Y | 42.48 | 0.35 | 0.45 | 0.05 | 0.36 |
| 8Y | 45.34 | 0.57 | 0.23 | 0.12 | 0.63 |
| 8Y | 45.23 | 0.12 | 0.13 | 0.25 | 0.38 |
| 13Y | 46.3 | 0.22 | 0.28 | 0.13 | 0.62 |
| 13Y | 43.83 | 0.70 | 0.62 | 0.35 | 0.68 |
| 13Y | 44.42 | 0.50 | 0.33 | 0.45 | 0.73 |
| 18Y | 44.34 | 0.42 | 0.48 | 0.32 | 0.72 |
| 18Y | 45.58 | 0.50 | 0.72 | 0.52 | 0.70 |
| 18Y | 47.41 | 0.72 | 0.59 | 0.59 | 0.81 |

**Table F**. Soil enzyme activities in wet season.

| Sites | β-glucosidase | Cellulase | Urease | Protease | Phosphatase |
| --- | --- | --- | --- | --- | --- |
| NH | 44.29 | 0.12 | 0.13 | 0.25 | 0.37 |
| NH | 45.33 | 0.25 | 0.29 | 0.04 | 0.42 |
| NH | 44.13 | 0.58 | 0.41 | 0.17 | 0.35 |
| 8Y | 44.92 | 0.20 | 0.53 | 0.10 | 0.53 |
| 8Y | 45.77 | 0.56 | 0.23 | 0.27 | 0.72 |
| 8Y | 46.29 | 0.42 | 0.34 | 0.14 | 0.57 |
| 13Y | 48.19 | 0.46 | 0.66 | 0.61 | 0.72 |
| 13Y | 48.68 | 0.75 | 0.56 | 0.46 | 0.73 |
| 13Y | 47.95 | 0.62 | 0.41 | 0.51 | 0.89 |
| 18Y | 50.41 | 0.61 | 0.73 | 0.76 | 0.77 |
| 18Y | 50.19 | 0.75 | 0.88 | 0.56 | 0.92 |
| 18Y | 49.27 | 0.70 | 0.56 | 0.59 | 0.88 |
